# Biochemical and Antioxidant Properties as well as Antimicrobial and Antibiofilm Activities of *Allium scorodoprasum* subsp. *jajlae* (Vved.) Stearn

**DOI:** 10.3390/cimb45060316

**Published:** 2023-06-07

**Authors:** Kerem Canli, Dilay Turu, Atakan Benek, Mustafa Eray Bozyel, Özcan Simsek, Ergin Murat Altuner

**Affiliations:** 1 Department of Biology, Faculty of Science, Dokuz Eylül University, Izmir 35390, Turkey; m.eraybozyel@gmail.com; 2 Fauna and Flora Research and Application Center, Dokuz Eylül University, Izmir 35390, Turkey; 3 Department of Biology, Graduate School of Natural and Applied Science, Dokuz Eylül University, Izmir 35390, Turkey; dilayturu@gmail.com; 4 Department of Biology, Graduate School of Natural and Applied Sciences, Kastamonu University, Kastamonu 37150, Turkey; atakan.benek@hotmail.com; 5 Department of Forestry, Yenice Vocational School, Çanakkale Onsekiz Mart University, Çanakkale 17950, Turkey; ozcansimsek@comu.edu.tr; 6 Department of Biology, Faculty of Science, Kastamonu University, Kastamonu 37150, Turkey; ergin.murat.altuner@gmail.com

**Keywords:** *Allium scorodoprasum* subsp. *jajlae*, antimicrobial activity, antioxidant activity, antibiofilm activity, GC-MS analysis

## Abstract

In this study, the chemical composition and biological activity of *Allium scorodoprasum* subsp. *jajlae* (Vved.) Stearn were investigated for the first time, focusing on its antimicrobial, antioxidant, and antibiofilm properties. A GC-MS analysis was employed to evaluate the composition of its secondary metabolites, identifying linoleic acid, palmitic acid, and octadecanoic acid 2,3-dihydroxypropyl ester as the major compounds in ethanol extract. The antimicrobial activity of *A. scorodoprasum* subsp. *jajlae* was assessed against 26 strains, including standard, food isolate, clinical isolate, and multidrug-resistant ones, as well as three *Candida* species using the disc diffusion method and the determination of the minimum inhibitory concentration (MIC). The extract showed strong antimicrobial activity against *Staphylococcus aureus* strains, including methicillin-resistant and multidrug-resistant strains, as well as *Candida tropicalis* and *Candida glabrata*. Its antioxidant capacity was evaluated using the DPPH method, revealing a high level of antioxidant activity in the plant. Additionally, the antibiofilm activity of *A. scorodoprasum* subsp. *jajlae* was determined, demonstrating a reduction in biofilm formation for the *Escherichia coli* ATCC 25922 strain and an increase in biofilm formation for the other tested strains. The findings suggest potential applications of *A. scorodoprasum* subsp. *jajlae* in the development of novel antimicrobial, antioxidant, and antibiofilm agents.

## 1. Introduction

Antimicrobial resistance undeniably stands as one of the most pressing and significant medical challenges of the 21st century. Recognizing its far-reaching implications, the World Health Organization has classified antimicrobial resistance as a major threat to both global health and food security [1]. Despite the development of effective antimicrobials over the years, the mounting resistance to existing antimicrobial agents has become increasingly concerning [2]. The emergence of multidrug resistance, predominantly among Gram-negative bacteria but also impacting all bacteria types, demands particular attention [3]. Consequently, the development of novel antimicrobial drugs is of paramount importance. Although the advent of antibiotics led to a decline in the use of plant-derived antimicrobials, microbiologists have recently renewed their interest in antimicrobial plant extracts for several reasons [4]. Plant materials, therefore, continue to serve as a vital and promising source of medicine in the global fight against severe diseases.

In recent years, there has been a worldwide trend toward natural phytochemicals, most commonly found in herbs, seeds, beans, fruits, vegetables, and teas [5]. The therapeutic value of plants comes from some chemical compounds that are active and have an impact on the body. Alkaloids, tannins, flavonoids, and phenolic compounds are the most significant of these bioactive plant substances [6]. Phenolic compounds have effectively been used to prevent pathogenic bacteria from growing and forming biofilms [7]. In addition to the identification of novel therapeutic molecules, these compounds naturally occurring in higher plants have long been known to have antioxidant activity. Recently, the biochemical actions of naturally occurring antioxidants and the role of oxygen-containing free radicals in biological systems have gained significant attention [8].

The antimicrobial activity and bioactive chemical compounds of *Allium* species have been known for a long time, exhibiting inhibitory effects against a wide range of pathogens, including bacteria, fungi, viruses, and parasites [9]. *Allium* is the most important among the 30 genera of the Alliaceae family. Plants of this genus form tubers or bulbs, and the flower stalks can reach heights from a few centimeters to one and a half meters [10,11]. Many *Allium* species, including economically significant ones such as onions, garlic, chives, and leeks, are still utilized today as vegetables, seasonings, and medicinal plants. Many species of *Allium* are also widely used in folk medicine for various health issues, such as hemorrhoids, hypertension, rheumatism, and hair loss [12,13,14]. Pharmacological investigations into *Allium scorodoprasum* tubers have revealed a range of bioactive properties, including antioxidant, antiviral, antifungal, antibacterial, anti-obesity, anti-hypertensive, diuretic, and anticancer effects [15].

The *Allium* genus presents considerable taxonomic challenges; despite *Allium scorodoprasum* subsp. *jajlae* being previously classified as an independent species under the designation *A. jajlae* and, in certain instances, categorized within alternative species, comprehensive taxonomic revisions have ultimately established its most accurate classification as a subspecies within *A. scorodoprasum* [16].

In this study, the antioxidant, antibiofilm, and antimicrobial activities of *A. scorodoprasum* subsp. *jajlae* samples collected from Samsun, Türkiye were investigated. The antibacterial activity of the ethanol extract against 23 bacteria and 3 yeast species was evaluated using the disc diffusion method. MIC tests were also performed on the microorganisms, which showed significant results. The antibiofilm activity was assessed on five bacteria previously identified to have biofilm formation. Moreover, a GC-MS analysis was employed to examine the samples’ volatile content and secondary metabolites. This research represents the first study on *A. scorodoprasum* subsp. *jajlae* samples in the literature.

## 2. Materials and Methods

### 2.1. Plant Sample

Dr. Mustafa Eray Bozyel collected and identified the plant samples of *A. scorodoprasum* subsp. *jajlae*, which were found in the region of Samsun, Turkey. Voucher specimens were stored at Dokuz Eylül University’s FAMER (Research and Application Center of Fauna Flora), under the personal herbarium sample number FFBDEU-ERC0018.

### 2.2. Extraction Process

Upon collection, all of the plants were dried and subsequently ground into a fine powder using a grinder, emphasizing the use of the whole plant. The powdered plant material was subjected to two separate agitation processes: one in ethanol (Sigma-Aldrich, St. Louis, MO, USA) and the other in water, both at 160 rpm for two days at ambient temperature. Following the agitation, the mixtures were filtered using Whatman No.1 filter paper to remove any remaining residues. The ethanol extract were subsequently evaporated using a rotary evaporator at 40 °C and the water extract was evaporated by a freeze dryer under vacuum [17]. The dried extracts were weighed, after which an extract stock was prepared. For disc diffusion, 50, 100, and 200 µL of this extract, corresponding to 0.9625, 1.925, and 3.85 mg, respectively, were applied onto empty, sterile antibiotic discs. For the MIC test, the plant sample was dissolved in DMSO, and DMSO was employed as a negative control at non-toxic concentrations (0.5 to 0.005%). The final DMSO and water solutions were filtered using a 0.45 µm filter.

Ethanol was used in antibacterial, antifungal, and antioxidant tests to determine the biochemical composition of the extract. Since this extract contains ethanol, it was replaced with water in order not to inhibit the growth of microorganisms in the antibiofilm study.

### 2.3. Microorganisms

In this study, a total of 23 bacterial strains were utilized, including 6 standard strains, 6 food isolates, 5 clinical isolates, and 6 multi-drug resistant strains. Additionally, the antifungal activity of the plant sample was tested against standard yeast *Candida albicans* DSMZ 1386 and clinically isolated yeasts, *Candida glabrata* and *Candida tropicalis*. The bacteria and yeast strains were obtained from the Department of Biology at Dokuz Eylül University’s Faculty of Science (Izmir, Türkiye).

### 2.4. Inoculum Preparation

All bacterial strains were incubated for 24 h at 37 °C, while *Candida albicans* DSMZ 1386, *Candida glabrata*, and *Candida tropicalis* were incubated for 48 h at 28 °C. Each inoculum was prepared separately by collecting morphologically similar colonies from nutrient agar by a sterile loop and suspended in 0.9% sterile saline solution. The turbidity of each inoculum was adjusted by comparing the 0.5 McFarland standard. After standardization of all the inocula, they were accepted to contain approximately 10^8^ CFU·mL^−1^ for bacteria and 10^7^ CFU·mL^−1^ for yeasts [17].

### 2.5. Antimicrobial Assay

The disc diffusion assay, as described by Andrews, was used to evaluate the antibacterial activity of *A. scorodoprasum* subsp. *jajlae* ethanol extract [18]. Mueller–Hinton agar was poured into 90 mm sterile Petri dishes to achieve an average depth of 4.0 ± 0.5 mm. Empty 6 mm antimicrobial susceptibility test discs were impregnated with the extracts. To eliminate potential solvent residue that could influence the outcome, the discs were dried at 30 °C for 24 h. The culture medium surfaces were then inoculated with microorganisms suspended in a saline solution. In aseptic conditions, the plates were allowed to dry for 5 min at room temperature before the discs were positioned onto the plates [17]. After incubation, the inhibitory zone sizes were measured and recorded. In the disc diffusion assay, empty sterile discs and the extraction solvent ethanol served as negative controls, while gentamicin was used as a positive control.

The minimum inhibitory concentration (MIC) values of the plant samples were determined using the broth microdilution technique [19]. Mueller–Hinton broth (MHB) was employed for cultivating various microbial strains. The cell density of each reference strain solution was adjusted to the 0.5 McFarland standard (1.5 × 10^8^ CFU/mL). A series of plant material dilutions were prepared, and a 100 µL sample from each dilution was transferred into 96-well sterile plates. Then, 50 µL of the microbial inocula was added to achieve a final volume of 100 µL in each well. Visual inspection was used to assess microbial growth. The positive control consisted of MHB inoculated with the test microorganisms. The MIC is the minimum concentration of plant sample necessary to inhibit bacterial growth after a 24-h incubation period. The results were reported in mg/mL following three repetitions of the tests.

### 2.6. Determination of Scavenging Activity on 1,1-diphenyl-2-picrylhydrazyl (DPPH) Radicals

This method evaluates the antioxidant capacity of the extract by measuring its ability to scavenge DPPH radicals. A total of 3.9432 mg of 2,2-diphenyl-1-picrylhydrazyl (DPPH) was accurately weighed and dissolved in 50 mL of ethanol to prepare the DPPH solution [8]. To protect the DPPH solution from light exposure, the exterior of the glass bottle was covered with aluminum foil. The extract was mixed with the DPPH solution, and the resulting mixture was allowed to stand at room temperature in the dark for 30 min. After incubation, the absorbance of the samples was measured at 515 nm using a spectrophotometer. Ascorbic acid was employed as a positive control in the antioxidant assay [20].

### 2.7. Antibiofilm Activity Assessment

The antibiofilm activity test used in this study was adapted from Karaca et al. [21] and consists of two stages: determining the conditions for biofilm formation and assessing the antibiofilm activity of *A. scorodoprasum* subsp. *jajlae* water extract.

For determining biofilm formation conditions, all strains were adjusted to 0.5 McFarland and added to media with glucose monohydrate ratios of 0%, 0.5%, 1.5%, 2%, and 2.5% for 24 and 48 h of culturing at 37 °C. After incubation, 200 μL of crystal violet was added to each well of the microplate, left for 15 min, and then washed with distilled water. Finally, 200 μL of an ethanol/acetone (70:30) solution was added to the wells, and after 15 min, the solution was transferred to clean microplates using a micropipette. Measurements were made at 550 nm on microplates. Following the protocols, high-biofilm-production conditions for *B. subtilis* DSMZ 1971, *L. monocytogenes* ATCC 7644, *E. coli* ATCC 25922, *L. innocua*, and *E. coli* (MDR) strains, which were selected according to previous unpublished studies, were determined as 48 h and a 1.5% glucose monohydrate concentration.

To assess antibiofilm activity, 100 μL of *A. scorodoprasum* subsp. *jajlae* water extract was added to the wells in row A of microplates containing media with a 1.5% glucose monohydrate concentration. The extract in the first well was diluted two-fold from row A to row G. Strains were transferred to microplates and standardized to 0.5 McFarland in sterile 0.9% saline solution before 48 h of incubation at 37 °C. Halamid was used as a positive control. After incubation, the procedures from the first step were performed on the microplates, and antibiofilm activities were determined by taking readings at 550 nm with a microplate reader device.

### 2.8. Gas Chromatography–Mass Spectrometry (GC-MS) Analysis

For many years, GC-MS has been employed as a technique to characterize the chemical composition of plants [22]. Gas chromatography separates the components within the mixture, while mass spectrometry aids in the structural identification of each component. In this study, an Agilent 8890 model GC/MS device with an HP-5 column was utilized. For the identification of chemical constituents, the analytical conditions included the following: an injector at 350 °C; carrier gas helium at 1.78 mL/min; injection mode: split, split ratio 10:1; volume injected: 1 µL of ethanol extract; and oven temperature programmed as follows: 40 °C to 150 °C at 4 °C/min, 150 °C to 180 °C at 3 °C/min, 180 °C to 230 °C at 2 °C/min, and 230 °C to 280 °C at 1 °C/min; pressure: 100 kPa; and total flow: 13.7 mL/min. MS scanning conditions included an interface temperature of 250 °C and an ion source temperature of 200 °C [17]. Using the GC/MS method, ions generated by electron ionization were separated according to their mass-to-charge ratios and recorded by the detector, with data obtained via computer. Compounds were identified by comparing data with the latest NIST and Wiley data libraries. 

### 2.9. Statistical Analysis

All experiments were conducted in triplicate. For the statistical analysis, a one-way ANOVA was employed, with a significance level set at 0.05. The Pearson correlation coefficient was calculated to assess the relationship between concentration and activity. All statistical analyses were performed using R Studio [23].

## 3. Results

The ethanol extract of *A. scorodoprasum* subsp. *jajlae* was tested against 26 strains to determine its antimicrobial and antifungal activity, and it exhibited activity against 12 strains. The extract showed antimicrobial activity against *S. aureus*, including standard, multi-drug resistant (MDR), and methicillin-resistant (MRSA) strains, as well as another *Staphylococcus* species, *S. epidermidis*. The extract was also found to be effective against two clinically isolated *Candida* yeast strains, *Candida tropicalis* and *Candida glabrata*. Furthermore, the extract demonstrated activity against two food isolate *Salmonella* strains, *S. infantis* and *S. kentucky*.

The antimicrobial and antifungal activity results are presented in the tables below, with the inhibition zone diameters given in millimeters and represented by the mean values of three replicates along with the standard errors. Table 1 shows the antimicrobial susceptibility of the standard strains, Table 2 displays the susceptibility of the food isolate strains, Table 3 presents the results for the clinical isolate strains, and Table 4 covers the susceptibility of the MDR strains, all of which were assessed by the disc diffusion method. No activity was observed for the negative controls. Furthermore, the statistical analysis revealed no significant differences between the outcomes of the three replicates for each extract concentration (*p* > 0.05).

The MIC values are presented in Table 5, and the plant extract exhibited the most potent effect against the four strains of the *Staphylococcus* genus, with MIC values of 4.81 and 9.62 mg/mL. The extract demonstrated a less pronounced impact on *S. mutans* compared to other *Staphylococcus* strains. Furthermore, the extract’s MIC value for the *Candida* species was determined to be 4.81 mg/mL. The weakest antibacterial effect was observed against *S. infantis*, although activity against the *Salmonella* species was also noted.

The antioxidant activity of *A. scorodoprasum* subsp. *jajlae* was assessed using the DPPH radical scavenging method, and the results are presented in Table 6. The DPPH radical scavenging assay revealed the EC_50_ value for *A. scorodoprasum* subsp. *jajlae* was 0.4940 mg/mL. The Pearson correlation analysis for the *A. scorodoprasum* subsp. *jajlae* DPPH scavenging activity revealed a very high positive correlation with a coefficient of r = 0.9783, indicating that the scavenging activity increases with the concentration.

In this study, the effects of *A. scorodoprasum* subsp. *jajlae* extract on the growth of the tested microorganisms’ biofilms were investigated, and their Pearson correlation coefficients were computed to determine the relationship between the biofilm formations and extract concentrations (Table 7). According to the Pearson correlation coefficients, it can be inferred that increasing the extract concentration enhances the production of biofilms for *L. innocua, B. subtilis* DSMZ 1971, and *E. coli* (MDR). On the other hand, a biofilm-inhibiting effect was observed for *E. coli* ATCC 25922.

The chemical composition of the *A. scorodoprasum* subsp. *jajlae* ethanol extract was analyzed using gas chromatography–mass spectrometry (GC-MS). The main components accounting for more than 0.5% of the chemical content were considered significant and are presented in Table 8. The GC-MS results revealed the presence of 28 distinct compounds in the plant extract, with linoleic acid, palmitic acid, and octadecanoic acid 2,3-dihydroxypropyl ester being the predominant components of the ethanol extract.

## 4. Discussion

Cardiovascular diseases remain the leading cause of mortality worldwide, accounting for 31% of all deaths, with infectious diseases ranking as the second most common cause of mortality at 17% [38]. *S. aureus* is a major human pathogen responsible for various illnesses, including life-threatening conditions such as pneumonia, endocarditis, osteomyelitis, skin and soft tissue infections, and bacteremia. As such, the development of novel antimicrobial agents effective against *Staphylococcus* species, which contribute to increased hospital mortality rates and pose significant public health threats, is of paramount importance [38]. Our findings are in line with those of previous studies reporting the antimicrobial activity of *Allium* species against *Staphylococcus* species [39,40].

The ethanol extract of *A. scorodoprasum* subsp. *jajlae* demonstrated significant antimicrobial activity against various *Staphylococcus* strains, including methicillin-resistant *S. aureus* (MRSA) and multidrug-resistant (MRSI + MDR) *S. aureus*, as well as *S. epidermidis* DSMZ 20044. This is particularly noteworthy given the increasing prevalence of drug-resistant infections and the associated public health concerns. The considerable antimicrobial activity displayed by the *A. scorodoprasum* subsp. *jajlae* extract against *S. aureus* strains, particularly the MRSA and MDR strains, highlights its potential as an alternative therapeutic agent against these multidrug-resistant pathogens.

*Salmonella* typically presents with fever, abdominal pain, nausea, diarrhea, and occasionally vomiting, with more severe complications in some cases. It is the most common microorganism responsible for foodborne infections [41]. In this study, effects were observed against three *Salmonella* strains, two of which were food isolates, highlighting the importance of prevention against these bacteria.

High mortality rates are attributed to fungi such as *Candida* species, which continue to be a significant health concern [42]. *A. scorodoprasum* subsp. *jajlae* extract demonstrated antifungal activity against two of the three tested *Candida* species. This effect is believed to be supported by the antifungal agent Elemicin, which was identified in the GC-MS analysis [30].

Reactive oxygen species (ROS) contribute to complications associated with oxidative stress, and the detrimental effects of ROS can be neutralized by antioxidants. As synthetic antioxidants have adverse effects on human health, scientists are seeking novel antioxidant sources without harmful consequences. The primary aim of the present study is to investigate the antioxidant activity of *A. scorodoprasum* subsp. *jajlae* using DPPH. It has been reported that *A. scorodoprasum* is a potent DPPH scavenger [43]. Additionally, methyleugenol and vanillin, identified in the GC-MS analysis, are known for their antioxidant activities.

Our findings on the antimicrobial, antioxidant, and antibiofilm properties of *A. scorodoprasum* subsp. *jajlae* differ in some aspects from those reported by Ðordevski et al. [37] for the closely related taxon, *A. scorodoprasum* L. flower extract. The chemical composition of *A. scorodoprasum* subsp. *jajlae* in our study, as determined by the GC-MS analysis, identified linoleic acid, palmitic acid, and octadecanoic acid 2,3-dihydroxypropyl ester as the major compounds in the ethanol extract. In contrast, Ðordevski et al. [44] found flavonoids, such as rutin and kaempferol 3-O-glucoside, to be the most abundant compounds in the extracts of *A. scorodoprasum* L. flowers.

The differences in chemical composition between our study on *A. scorodoprasum* subsp. *jajlae* and Ðordevski et al.’s study [44] on *A. scorodoprasum* L. flowers could stem from several factors. One contributing factor is the variation in plant material, as our study investigated the whole plant of *A. scorodoprasum* subsp. *jajlae*, while Ðordevski et al. focused on the flowers of the closely related but distinct taxon, *A. scorodoprasum* L. Additionally, variations in extraction solvents and environmental conditions may also influence the differences observed in the chemical composition of the respective plant extracts.

Our investigation of the antimicrobial activity of *A. scorodoprasum* subsp. *jajlae* against various bacterial and *Candida* species revealed strong activity against *S. aureus* strains, including methicillin-resistant and multidrug-resistant strains, as well as *C. tropicalis* and *C. glabrata*. In comparison, Ðordevski et al. [44] found that ethanol-containing and water extracts were more efficient against fungi, while butanol-containing extracts had a better effect on the growth inhibition of bacteria. Furthermore, their study reported significant antibiofilm activity against *Staphylococcus lugdunensis* biofilms. These distinctions between the two studies’ findings may provide valuable insights for future research into the development of novel antimicrobial, antioxidant, and antibiofilm agents derived from *A. scorodoprasum* and its subspecies.

According to Mollica et al. [43], the flower extract of *A. scorodoprasum* exhibited a higher scavenging activity of 34.83 mg TE/g extract on DPPH radicals. In comparison, as shown in Table 6, the scavenging activity of *A. scorodoprasum* subsp. *jajlae* extract increased with increases in concentration, reaching a maximum of 38.118% at 200 µg/mL. This difference in antioxidant activity could be attributed to variations in the phenolic content between the two subspecies. The flower extract of *A. scorodoprasum* was found to be rich in phenolic compounds, including rosmarinic acid, p-hydroxybenzoic acid, and protocatechuic acid [43]. These compounds have been reported to exhibit antioxidant activity in various assays. In contrast, the phenolic composition of *A. scorodoprasum* subsp. *jajlae* might differ, leading to variations in the observed antioxidant activities.

Dehpour et al. [45] reported on the chemical composition and antibacterial properties of essential oils obtained from the flowers of *A. rotundum* L., an important wild *Allium* species in Iran. Their GC-MS analysis identified 54 compounds, representing 68% of the total oil. The major compounds identified were different from those found in our study, which focused on *A. scorodoprasum* subsp. *jajlae*. Moreover, the antibacterial activity of *A. rotundum* essential oils was evaluated against *Proteus mirabilis*, *Enterobacter cloacae*, *Klebsiella pneumonia*,e *Staphylococcus aureus*, and *Bacillus subtilis*, and showed inhibition at concentrations of <1/200 (*v*/*v*). In our study, *A. scorodoprasum* subsp. *jajlae* ethanol extract displayed antimicrobial activity against several bacteria, including *Staphylococcus*, *Salmonella*, and *Candida* species. The differences in the chemical compositions and antimicrobial activities between the two *Allium* species highlight the importance of investigating various *Allium* species and their potential applications in combating infectious diseases.

The study findings revealed that 12 of the identified compounds have biological activity, as previously reported in other research. The observed antibacterial, antifungal, and antioxidant activities are supported by compounds with known properties. Further tests at the compound level are required to determine the effects of substances for which no known effect exists and to establish whether the compounds producing the observed effects act alone or in combination with other substances. Based on the results obtained from the study, it is not possible to determine the potency of the observed effects due to the fact that they originate from the extracts rather than isolated compounds. Therefore, in future studies, it is essential to perform purification processes to individually assess the potency of each compound, ensuring a comprehensive understanding of their activity. This approach will contribute to the overall understanding of the mechanisms and potential applications of these compounds in various settings.

In the field of systematics, conducting studies on the biochemical composition and bioactivity of species that have faced or may face challenges in the past or future can indirectly shed light on their systematic research. By doing so, these studies will provide valuable data that can be utilized to reveal the differences among species and support the understanding of their distinctions in a more comprehensive manner. While the primary focus of this research is on biochemical composition and bioactivity effects, it also offers a secondary benefit by indirectly contributing to the advancement of systematics.

## 5. Conclusions

This study demonstrated the potential antimicrobial, antifungal, and antioxidant activities of *A. scorodoprasum* subsp. *jajlae* ethanol extract against some microorganisms, including standard, food isolate, and multidrug-resistant strains. The observed activities are supported by the presence of biologically active compounds identified through a GC-MS analysis. However, further research is necessary to elucidate the exact mechanisms behind these observed effects. Future studies should focus on the purification of individual compounds, as well as determining whether the observed activities are a result of single compounds or synergistic interactions between multiple constituents. This information will contribute to a better understanding of the potential therapeutic applications of *A. scorodoprasum* subsp. *jajlae* and its constituents in the development of novel antimicrobial, antifungal, and antioxidant agents.

## Figures and Tables

**Table 1:** Antimicrobial susceptibility of standard strains assessed by disc diffusion method.

Microorganisms	50 µL	100 µL	200 µL	r-Value *	GEN (10 µg)
*Candida albicans* DSMZ 1386	0.00 ± 0.00	0.00 ± 0.00	0.00 ± 0.00	-	12
*Escherichia coli* ATCC 25922	0.00 ± 0.00	0.00 ± 0.00	0.00 ± 0.00	-	22
*Listeria monocytogenes* ATCC 7644	0.00 ± 0.00	0.00 ± 0.00	0.00 ± 0.00	-	28
*Pseudomonas aeruginosa* DSMZ 50071	0.00 ± 0.00	0.00 ± 0.00	0.00 ± 0.00	-	15
*Salmonella enteritidis* ATCC 13076	0.00 ± 0.00	8.00 ± 0.00	8.00 ± 0.58	0.756	21
*Staphylococcus aureus* ATCC 25923	8.00 ± 0.00	11.00 ± 0.58	12.00 ± 0.58	0.891	21
*Staphylococcus epidermidis* DSMZ 20044	9.00 ± 0.58	10.00 ± 0.00	11.00 ± 0.00	0.982	22

Data presented as mean values ± standard errors (inhibition zone diameters in mm); * Pearson correlation coefficient; GEN: Gentamicin; DSMZ: Deutsche Sammlung von Mikroorganismen und Zellkulturen; ATCC: American Type Culture Collection.

**Table 2:** Antimicrobial susceptibility of food isolate strains assessed by disc diffusion method.

Microorganisms	50 µL	100 µL	200 µL	r-Value *	GEN (10 µg)
*Enterococcus durans*	0.00 ± 0.00	0.00 ± 0.00	0.00 ± 0.00	-	11
*Enterococcus faecium*	0.00 ± 0.00	7.00 ± 0.00	7.00 ± 0.00	0.756	28
*Klebsiella pneumoniae*	0.00 ± 0.00	0.00 ± 0.00	0.00 ± 0.00	-	19
*Listeria innocua*	0.00 ± 0.00	7.00 ± 0.00	8.00 ± 0.00	0.826	13
*Salmonella infantis*	8.00 ± 0.00	8.00 ± 0.00	8.00 ± 0.58	-	17
*Salmonella kentucky*	7.00 ± 0.00	8.00 ± 0.00	9.00 ± 0.58	0.982	12

Data presented as mean values ± standard errors (inhibition zone diameters in mm); * Pearson correlation coefficient; GEN: Gentamicin.

**Table 3:** Antimicrobial susceptibility of clinical isolate strains assessed by disc diffusion method.

Microorganisms	50 µL	100 µL	200 µL	r-Value *	GEN (10 µg)
*Streptococcus mutans*	8.00 ± 0.00	10.00 ± 0.58	11.00 ± 0.58	0.882	22
*Staphylococcus lugdunensis*	0.00 ± 0.00	0.00 ± 0.00	0.00 ± 0.00	-	17
*Acinetobacter baumannii*	0.00 ± 0.00	0.00 ± 0.00	0.00 ± 0.00	-	18
*Shigella flexneri*	0.00 ± 0.00	0.00 ± 0.00	0.00 ± 0.00	-	16
*Klebsiella pneumoniae*	0.00 ± 0.00	0.00 ± 0.00	0.00 ± 0.00	-	18
*Candida tropicalis*	8.00 ± 0.00	11.00 ± 0.00	13.00 ± 0.00	0.954	-
*Candida glabrata*	10.00 ± 0.00	10.00 ± 0.58	11.00 ± 0.00	0.837	7

Data presented as mean values ± standard errors (inhibition zone diameters in mm) * Pearson correlation coefficient; GEN: Gentamicin.

**Table 4:** Antimicrobial susceptibility of MDR strains assessed by disc diffusion method.

Microorganisms	50 µL	100 µL	200 µL	r-Value *	GEN (10 µg)
*Klebsiella pneumoniae*	0.00 ± 0.00	0.00 ± 0.00	0.00 ± 0.00	-	8
*Acinetobacter baumannii*	0.00 ± 0.00	0.00 ± 0.00	0.00 ± 0.00	-	15
*Streptococcus pneumonia*	0.00 ± 0.00	0.00 ± 0.00	0.00 ± 0.00	-	11
*Staphylococcus aureus* MRSA	11.00 ± 0.00	12.00 ± 0.00	12.00 ± 0.58	0.803	10
*Staphylococcus aureus* MRSA + MDR	10.00 ± 0.00	10.00 ± 0.58	10.00 ± 0.58	0.285	-
*Providencia rustigianii*	0.00 ± 0.00	0.00 ± 0.00	0.00 ± 0.00		22

Data presented as mean values ± standard errors (inhibition zone diameters in mm) * Pearson correlation coefficient; GEN: Gentamicin; MRSA: Methicillin-resistant *S. aureus*; MDR: Multi-drug resistance.

**Table 5:** Minimum inhibitory concentration (MIC) test results.

Microorganisms	MIC (mg/mL)
*Salmonella enteritidis* ATCC 13076	9.62
*Staphylococcus aureus* ATCC 25923	4.81
*Staphylococcus epidermidis* DSMZ 20044	4.81
*Enterococcus faecium* (FI)	.
*Listeria innocua* (FI)	.
*Salmonella infantis* (FI)	19.25
*Salmonella kentucky* (FI)	9.62
*Staphylococcus aureus* MRSA	4.81
*Staphylococcus aureus* MRSA+MDR	4.81
*Streptococcus mutans* (CI)	9.62
*Candida tropicalis* (CI)	4.81
*Candida glabrata* (CI)	4.81

CI: Clinical isolate, FI: Food isolate, MDR: Multidrug resistance, MRSA: Methicillin resistance *S. aureus.*

**Table 6:** DPPH radical scavenging activity results.

Concentration (µg/mL))	ASJ (%)	AA (%)
200.000	38.118	94.665
100.000	20.781	93.391
50.000	14.430	92.077
25.000	13.142	90.086
12.500	5.692	69.943
6.250	4.507	35.794
3.125	1.430	17.698
1.075	0.896	8.739

ASJ: *A. scorodoprasum* subsp. *jajlae* scavenging AA: Ascorbic acid scavenging.

**Table 7:** Pearson Correlation Coefficients for Antibiofilm Activity.

Microorganisms	Pearson Correlation Coefficient
*E. coli* (MDR)	0.923479464
*E. coli* ATCC 25922	−0.534721723
*L. innocua*	0.973313903
*L. monocytogenes*	0.264206475
*B. subtilis* DSMZ 1971	0.740007835

**Table 8:** GC-MS Analysis of *A. scorodoprasum* subsp. *jajlae* Ethanol Extract Components.

No	RT	Chemical Structures	Compound Name	Formula	MW (g/mol)	Area (%)	Known Activity
1	20.943	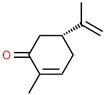	D-Carvone	C_10_H_14_O	150.218	0.58	Anti-inflammatory and protective effects [24]
2	23.502	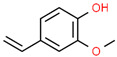	2-Methoxy-4-vinylphenol	C_9_H_10_O_2_	150.174	0.61	Anti-inflammatory effect [25]
3	26.375	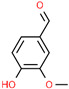	Vanillin	C_8_H_8_O_3_	152.147	0.66	Anticancer and antioxidant activity [26,27]
4	26.462	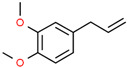	Methyleugenol	C_11_H_14_O_2_	178.228	1.02	Antioxidant, antimicrobial, genotoxicity, and herbicidal activities [28]
5	29.892	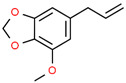	Myristicin	C_11_H_12_O_3_	192.211	3.15	Insecticidal activity [29]
6	31.139	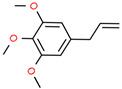	Elemicin	C_12_H_16_O_3_	208.254	1.09	Antiviral and antifungal activity [30,31]
7	37.749	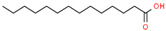	Myristic acid	C_14_H_28_O_2_	228.371	1.12	Antiviral activity [32]
8	39.746	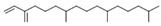	Neophytadiene	C_20_H_38_	278.516	1.32	Anti-inflammatory activity [33]
9	39.988	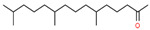	Hexahydrofarnesyl acetone	C_18_H_36_O	268.478	0.89	-
10	42.317	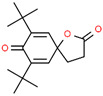	7,9-di-tert-butyl-1-oxaspiro [4.5]deca-6,9-diene-2,8-dione	C_17_H_24_O_3_	276.371	0.56	-
11	44.384	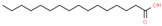	Palmitic acid	C_16_H_32_O_2_	256.424	15.01	Antitumor activity [34]
12	48.129	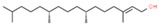	Phytol	C_20_H_40_O	296.531	1.66	Antimycobacterial activity [35]
13	49.504	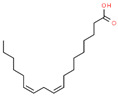	Linoleic acid	C_18_H_32_O_2_	280.445	25.77	Carcinogenesis and proliferative activity [36]
14	50.245	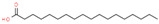	Stearic acid	C_18_H_36_O_2_	284.477	3.68	Synthesis, antidepressant, and antimicrobial activities [37]
15	56.502	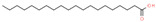	Arachidic acid	C_20_H_40_O_2_	312.530	1.14	-
16	60.745	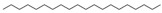	Icosane	C_20_H_42_	282.547	1.16	-
17	61.185	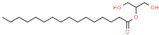	2-Palmitoylglycerol	C_19_H_38_O_4_	330.503	1.32	-
18	67.583	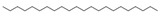	Docosane	C_22_H_46_	310.601	6.30	-
19	68.335	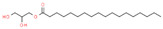	Octadecanoic acid 2,3-dihydroxypropyl ester	C_21_H_42_O_4_	358.556	7.23	-
20	74.184	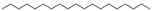	Nonadecane	C_19_H_40_	268.521	6.11	-
21	78.647	-	Unknown	-	-	0.71	-
22	80.431	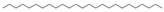	Pentacosane	C_25_H_52_	352.680	4.68	-
23	83.409	-	Unknown	-	-	0.88	-
24	85.552	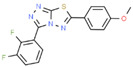	3-(2,3-Difluorophenyl)-6-(4-methoxyphenyl)[1,2,4]triazolo[3,4-b][1,3,4]thiadiazole	C_16_H_10_F_2_N_4_OS	344.339	0.58	-
25	86.414	-	Unknown	-	-	3.82	-
26	88.691	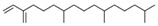	Neophytadiene	C_20_H_38_	278.5	0.92	-
27	90.932	-	Unknown	-	-	1.41	-
28	94.693	-	Unknown	-	-	1.04	-
Figures: https://pubchem.ncbi.nlm.nih.gov/ (accessed on 10 February 2023).							

## Data Availability

All data that were generated or analyzed during this study have been included in this published article.
